# Sex differences in the three-dimensional morphology of unruptured intracranial aneurysms

**DOI:** 10.1016/j.ynirp.2026.100328

**Published:** 2026-02-16

**Authors:** Maarten J. Kamphuis, Phebe J. Groenheide, Laura T. van der Kamp, Margot van Genderen, Ruben P.A. van Eijk, Jeroen Hendrikse, Gabriel J.E. Rinkel, Mervyn D.I. Vergouwen, Ynte M. Ruigrok, Irene C. van der Schaaf

**Affiliations:** a Department of Radiology, University Medical Center Utrecht, Utrecht University, Utrecht, the Netherlands; b Department of Neurology and Neurosurgery, UMC Utrecht Brain Center, University Medical Center Utrecht, Utrecht University, Utrecht, the Netherlands; c Biostatistics & Research Support, Julius Center for Health Sciences and Primary Care, University Medical Center Utrecht, Utrecht, the Netherlands

**Keywords:** Computed tomography angiography, Intracranial aneurysm, Magnetic resonance angiography, Radiomics, Sex, Subarachnoid hemorrhage

## Abstract

**Objective:**

Women have a higher rupture risk of intracranial aneurysms than men. One possible explanation is that aneurysm volume and shape irregularity, both linked to rupture risk, may differ between women and men. We investigated this with 3-dimensional morphological aneurysm parameters.

**Methods:**

In a random sample of consecutive patients with unruptured intracranial aneurysms diagnosed between 2008 and 2018, we quantified volume and shape parameters describing global (sphericity, elongation, and flatness) and local shape (shape index and curvedness) on CT or MR angiography scans. We compared these parameters between women and men (reference) with univariable and multivariable linear regression analysis (global parameters) and binary logistic regression analysis (local parameters). The multivariable analysis was adjusted for the confounders: age, hypertension, smoking status, aneurysm size, location, and imaging modality.

**Results:**

We included 326 patients (239 women [73%], mean age 58 years [SD 12 years]). Women had smaller aneurysm volumes than men in multivariable analysis (β −0.30 SD, 95%CI -0.55 to −0.06). The global shape parameters sphericity, elongation, and flatness were comparable between sexes. Of the local shape parameters, women had higher aneurysm shape index (OR 2.38, 95%CI 1.03 to 5.49) and lower curvedness (OR 0.50, 95%CI 0.29 to 0.88) in multivariable analysis.

**Conclusions:**

Women have intracranial aneurysms with smaller volumes and shape characteristics associated with lower rupture risk. Therefore, factors other than aneurysm morphology are likely responsible for the higher risk of aneurysm rupture in women.

## Highlights

• Women had smaller intracranial aneurysm (IA) volumes than men.
• Women showed higher IA shape index and lower curvedness than men.
• 3D IA morphology did not explain higher rupture risk in women.
• IA research should adjust or stratify analyses by sex.

## Graphical abstract

**Figure:**
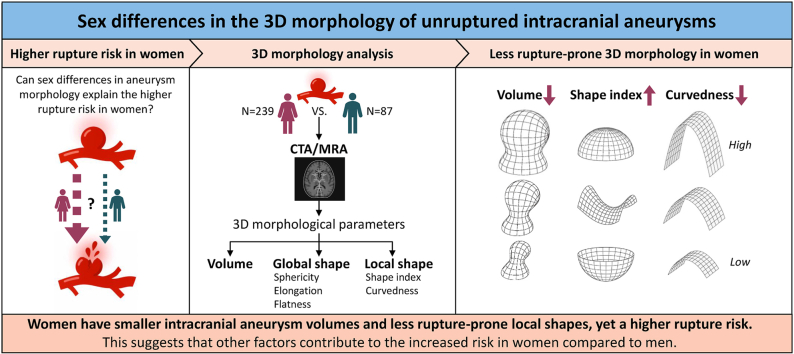

## 1 Introduction

Approximately 3% of the general population has an unruptured intracranial aneurysm (UIA) (Vlak et al., 2011). Rupture of an aneurysm leads to subarachnoid hemorrhage (SAH), a subtype of stroke associated with high rates of mortality and morbidity (Rehman et al., 2022; Asikainen et al., 2023). The incidence of SAH is higher among women (Etminan et al., 2019), which may be attributed in part to a higher prevalence of aneurysms in women compared to men (Vlak et al., 2011). However, even among patients with UIAs, women have a higher risk of rupture compared to men (Zuurbier et al., 2022), also after adjustment for various patient and aneurysm characteristics such as age, hypertension, smoking status, family history, aneurysm size and aneurysm location (Zuurbier et al., 2022). One factor that could account for the higher rupture risk in women, which was not adjusted for in the previous study, is aneurysm morphology. Larger aneurysms with an irregular shape are at increased risk of aneurysm growth and rupture (Greving et al., 2014; Backes et al., 2017; Kleinloog et al., 2018). Previous studies did not find sex differences in aneurysm size and shape (Krzyżewski et al., 2018; Wälchli et al., 2024). Size was measured with manual linear measurements, which consider only two dimensions of the aneurysm (Krzyżewski et al., 2018; Wälchli et al., 2024), and shape assessments were conducted through visual inspection, categorizing aneurysms as regular or irregular based on the absence or presence of blebs or multiple lobes (Wälchli et al., 2024). Technological advances now allow for the quantified assessment of aneurysm morphology using 3-dimensional (3D) parameters, improving the ability to identify detailed relationships between sex and morphology (Timmins et al., 2022). In this cross-sectional study, we investigated with 3D morphological parameters whether aneurysm volume and shape are different in women compared to men, while adjusting for relevant confounders.

## 2 Methods

### 2.1 Study population

The institutional review board of University Medical Center (UMC) Utrecht, the Netherlands, waived the requirement for formal ethical assessment and informed consent of this cross-sectional study, because data from routine patient care were used (NedMec, 22-737). Patients were retrieved retrospectively from the institutional database of UMC Utrecht. This database consists of all patients who were diagnosed with an intradural UIA between 2008 and 2018. We drew a computer-generated random sample without replacement from this database (sampling rate 50%) and screened the resulting sample for eligibility. We excluded patients with non-saccular aneurysms, intraluminal thrombus, large calcifications on CT angiography that hindered morphological measurements, insufficient image quality, and those who opted out of data use for research purposes. A computer-generated random sample was drawn because manual aneurysm segmentation is highly labor-intensive. Based on the a priori sample size calculation, this approach provided sufficient statistical power while eliminating selection bias.

### 2.2 Collection of patient and aneurysm characteristics

Patient data were collected from medical records. History of hypertension was defined as a physician-diagnosed condition or use of antihypertensive medications. Smoking status was categorized as current, former (if stopped at least 3 months before diagnosis), or never. Aneurysm location was extracted from radiology reports, and reviewed during annotation of the aneurysms. Aneurysm location was categorized as anterior cerebral or communicating artery, internal carotid artery, posterior communicating artery, middle cerebral artery, or posterior circulation. Aneurysm size was measured on multiplanar reconstructions of the aneurysm.

### 2.3 Three-dimensional morphological measurements

Diagnostic CT angiography or time-of-flight MR angiography (TOF MRA) was used for the 3D morphological measurements that were performed as described elsewhere (Timmins et al., 2022). To harmonize differences in imaging protocols and resolution, all scans were preprocessed and resampled to an identical voxel size. A researcher with >3 years of experience (M.J.K.) who was blinded for patient characteristics annotated aneurysms by drawing contours around the aneurysm on axial slices using in-house developed software implemented in MeVisLab (MeVis Medical Solutions). The software fitted a mesh around the aneurysm using a marching cubes algorithm. Based on this mesh, volume and surface area, along with three global shape parameters (sphericity, elongation, and flatness), were calculated in accordance with the Imaging Biomarker Standardization Initiative (Zwanenburg et al., 2020). Sphericity is derived from volume and surface area and indicates the deviation from a perfect sphere, with lower values signifying a greater deviation. Elongation is based on the ratio of the minor to major axis length, with lower values indicating more elongated shapes. Flatness is based on the ratio of the least to major axis length, where lower values represent flatter shapes. In addition, two local aneurysm shape parameters were calculated: shape index and curvedness. Shape index describes how concave (low values) or convex (high values) a shape is; curvedness describes how strong the curvature is, with high values indicating strong curvatures (Koenderink and Doorn, 1992). These two parameters were calculated for each point on the mesh, and the median was calculated for further analyses.

### 2.4 Rupture-prone aneurysm morphology

Whether high or low values of volume and the global shape parameters were associated with rupture-prone aneurysms was based on a recent meta-analysis (Johnson et al., 2024). The meta-analysis found that larger volume and lower values of sphericity, elongation, and flatness are associated with aneurysm instability (Johnson et al., 2024). Shape index and curvedness were not part of this meta-analysis. Since low shape index values reflect surface concavities and high curvedness values reflect areas with more pronounced curvatures, these features indicated a rupture-prone shape (Koenderink and Doorn, 1992; Timmins et al., 2022).

### 2.5 Statistical analysis

Linear regression models were used to estimate the mean women-to-men difference in volume and global shape parameters (sphericity, elongation, and flatness). Volume and shape parameters were entered as dependent variable and sex as independent variable. In the multivariable analysis, we adjusted for the following confounders that have been associated with aneurysm morphology in the existing literature: age (Sanchez et al., 2023), hypertension (Liu et al., 2019), smoking status (Zhang et al., 2021), aneurysm size (Juchler et al., 2020), and aneurysm location (Juchler et al., 2020) (the model for volume was not adjusted for size). Imaging modality was additionally included as a covariate. Distributions of morphological parameters and normality of model residuals were assessed visually using histograms and Q-Q plots (Supplemental Figs. 1–3). We applied a natural log-transformation to volume and a Box-Cox transformation to elongation. All morphological parameters were subsequently scaled with Z-standardization (($X\;–\;\bar{X}$)/SD) to facilitate the comparison of sex differences among morphological parameters. Due to the low variability in local shape parameters (shape index and curvedness), these were dichotomized based on the median and analyzed with a logistic regression model. Exploratory analyses additionally analyzed shape index and curvedness as continuous outcomes to evaluate consistency of sex differences. Results were presented as regression coefficients (β) with 95% confidence interval (CI) for linear regression and odds ratio (OR) with 95% CI for binary logistic regression, using men as the reference category. For aneurysm location, the posterior communicating artery and posterior circulation were combined into a single category, consistent with their risk classification (Greving et al., 2014; Backes et al., 2017). For smoking, former and current smokers were combined into a single category. In case a patient had multiple aneurysms, the largest aneurysm was selected for analysis in accordance with previous studies (Greving et al., 2014; Zuurbier et al., 2022). Smoking history was missing for 7 of 326 patients (2%) and was assumed to be missing at random. To address this, we applied multiple imputation using the mice package implemented in R (Buuren and Groothuis-Oudshoorn, 2011), with 15 imputations and 30 iterations. The analyses were conducted on each imputed dataset, and results were combined using Rubin's rules (Rubin and Schenker, 1991).

To investigate the impact of the missing data for smoking status on the analyses, we performed two sensitivity analyses. In the first sensitivity analysis, we removed smoking as a covariable. In the second sensitivity analysis, we included smoking status as a covariable, but we only included the subset of patients with complete data for smoking status. All analyses were performed using R version 4.2.1 with the packages mice, sjmisc, dplyr, MASS, stringr, ggplot2, and ggpubr.

### 2.6 Sample size calculation

We performed a sample size calculation for multivariable linear regression analysis with 8 independent variables (age, hypertension, smoking status, aneurysm size, and aneurysm location [3 locations, 1 reference location], and sex), assuming an alpha of 5%, power of 80%, and an effect size of 0.05. The effect size is the proportion of variance explained by predictors, and 0.05 is considered small (Cohen, 2013). The sample size calculation yielded a minimum sample size of 307 patients.

## 3 Results

**Fig. 1:**
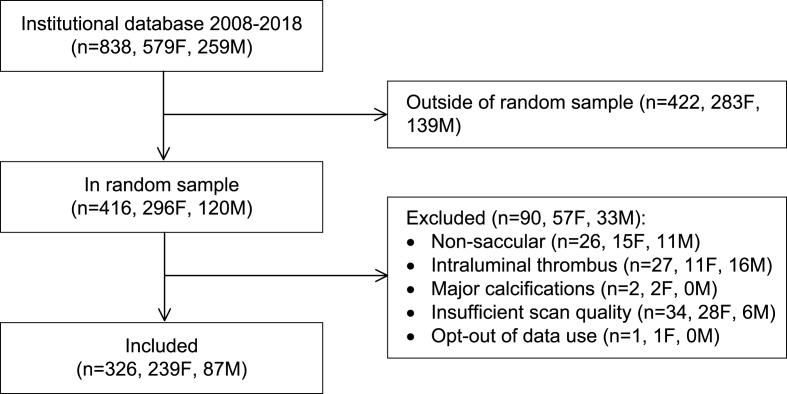
Patient flowchart. *F = female; M = male.*

The random sample consisted of 416 patients, of whom 90 were excluded (Fig. 1). The remaining 326 patients were included, of whom 239 (73%) were women, with a mean age of 58 years (SD 12) (Table 1). The median aneurysm size was 5.4 mm (interquartile range [IQR] 3.5–8.0) in women and 6.3 mm (IQR 4.5–9.3) in men.

**Table 1:** Patient and aneurysm characteristics.

	Total (n = 326)	Women (n = 239, 73%)	Men (n = 87, 27%)
**Patient characteristics**			

Mean age (SD)	58 (12)	58 (12)	61 (13)
Hypertension (%)	185 (57)	126 (53)	59 (68)
Stroke/TIA (%)	58 (18)	33 (14)	25 (29)
Previous SAH from another aneurysm (%)	68 (21)	59 (25)	9 (10)
Multiple aneurysms (%)	134 (41)	107 (45)	27 (31)
Smoking^a^ (%)			
Current	139 (43)	106 (44)	33 (38)
Former^b^	103 (32)	69 (29)	34 (39)
Never	84 (26)	64 (27)	20 (23)

**Aneurysm characteristics**			

Median size in mm (IQR)	5.8 (3.7–8.2)	5.4 (3.5–8.0)	6.3 (4.5–9.3)
Location (%)			
ACA/ACom	67 (21)	40 (17)	27 (31)
ICA	61 (19)	50 (21)	11 (13)
PCom	26 (8)	19 (8)	7 (8)
MCA	122 (37)	90 (38)	32 (37)
Posterior circulation	50 (15)	40 (17)	10 (12)
Imaging modality (%)			
MRA	132 (41)	98 (41)	34 (39)
CTA	194 (60)	141 (59)	53 (61)

ACA = anterior cerebral artery (including pericallosal artery); ACom = anterior communicating artery; CTA = CT angiography; ICA = internal carotid artery; IQR = interquartile range; MCA = middle cerebral artery; MRA = MR angiography; PCom = posterior communicating artery; SAH = subarachnoid hemorrhage; SD = standard deviation; TIA = transient ischemic attack.

a Data on smoking status were imputed for 7 of 326 patients (2%): 6 of 239 women (3%) and 1 of 87 men (1%).

b Patients were considered former smokers if they stopped smoking at least 3 months before aneurysm diagnosis.

**Fig. 2:**
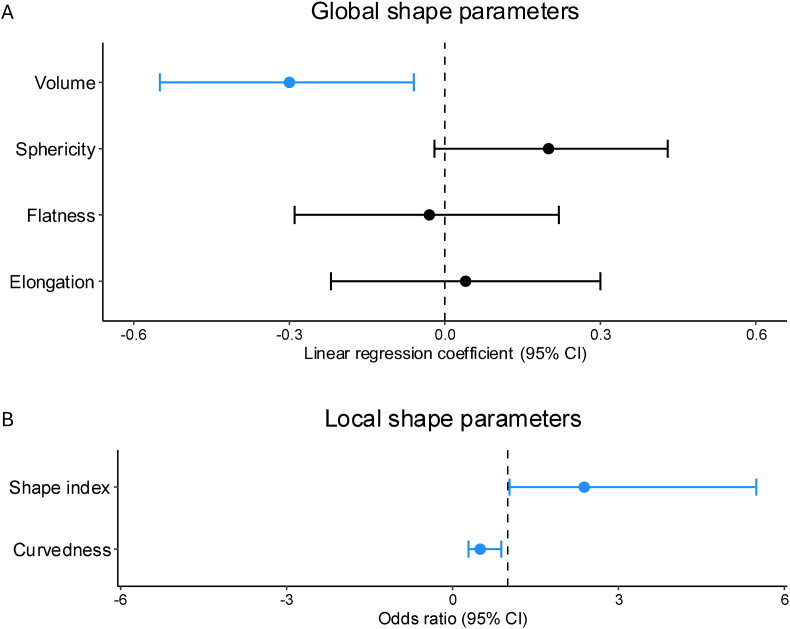
Forest plots showing women-to-men differences of morphological parameters in multivariable regression analysis. Scaled morphological parameters were used as dependent variables; sex and confounders (age, hypertension, smoking status, aneurysm size, aneurysm location, and imaging modality) as independent variables. Aneurysm volume was not adjusted for aneurysm size. Men were used as reference. **a** Global shape parameters were analyzed with linear regression. Women had lower aneurysm volume. Other global shape parameters did not differ. **b** Local shape parameters were analyzed with binary logistic regression; both parameters were less rupture-prone in women. Blue bars indicate less rupture-prone morphology characteristics; black bars indicate no statistically significant difference.

Women had a lower aneurysm volume than men in univariable analysis (β −0.33 SD, 95% CI -0.58 to −0.09) and in multivariable analysis (β −0.30 SD, 95% CI -0.55 to −0.06) (Table 2, Fig. 2, and Supplemental Fig. 4). Aneurysm sphericity was higher in women in univariable (β 0.38 SD, 95% CI 0.14 to 0.62), but not in multivariable analysis (β 0.20 SD, 95% CI -0.02 to 0.43). Aneurysm elongation and flatness were comparable between women and men. Aneurysm shape index was higher in women in univariable (OR 2.38, 95% CI 1.43 to 3.97) and multivariable analysis (OR 2.38, 95% CI 1.03 to 5.49). Aneurysm curvedness was comparable between women and men in univariable analysis (OR 0.67, 95% CI 0.41 to 1.09), but was lower in women in multivariable analysis (OR 0.50, 95% CI 0.29 to 0.88). In exploratory analyses treating the local shape parameters as continuous outcomes, women had a higher shape index than men in univariable analysis (β 0.37, 95% CI 0.12 to 0.61), but not after multivariable adjustment (β 0.06, 95% CI -0.07 to 0.20). For curvedness, a trend toward lower values in women compared to men was observed in univariable analysis (β −0.20, 95% CI -0.44 to 0.05), which became statistically significant after adjustment for confounders in the multivariable analysis (β −0.27, 95% CI -0.51 to −0.03). All sex differences in the 3D morphological parameters we found indicated less rupture-prone aneurysm morphology in women compared to men.

**Table 2:** Women-to-men differences in 3D quantified morphological parameters.

Global shape parameters					
	Median (interquartile range)			Women-to-men difference (β, 95% CI)^a^	
	Total (n = 326)	Women (n = 239)	Men (n = 87)	Univariable	Multivariable
Volume (mm^3^)	77 (25–246)	68 (21–207)	120 (36–305)	**−0.33 (-0.58 to -0.09)**^b^	**−0.30 (-0.55 to -0.06)**
Sphericity^c^	79 (76–82)	79 (76–82)	77 (74–80)	**0.38 (0.14 to 0.62)**^d^	0.20 (−0.02 to 0.43)
Elongation^c^	88 (83–93)	88 (83–93)	89 (82–92)	0.05 (−0.19 to 0.30)	0.04 (−0.22 to 0.30)
Flatness^c^	81 (76–85)	81 (75–84)	80 (76–86)	−0.08 (−0.32 to 0.17)	−0.03 (−0.29 to 0.22)

Local shape parameters							
	Median (interquartile range)			Women-to-men difference (β, 95% CI)^a^		Women-to-men difference (odds ratio, 95% CI)^d^	
	Total (n = 326)	Women (n = 239)	Men (n = 87)	Univariable	Multivariable	Univariable	Multivariable
Shape index^c^	32 (20–50)	34 (21–50)	31 (14–49)	**0.37 (0.12 to 0.61)**	0.06 (−0.07 to 0.20)	**2.38 (1.43 to 3.97)**^e^	**2.38 (1.03 to 5.49)**
Curvedness^c^	240 (235– 260)	240 (233– 258)	240 (240– 263)	−0.20 (−0.44 to 0.05)	**−0.27 (-0.51 to -0.03)**	0.67 (0.41 to 1.09)	**0.50 (0.29 to 0.88)**

Morphological parameters were used as dependent variables; sex and confounders (age, hypertension, smoking status, aneurysm size, aneurysm location, and imaging modality) as independent variables. Volume was not adjusted for aneurysm size. Men were used as reference. Statistically significant associations indicated in bold. Results are based on pooled estimates from 15 multiply imputed datasets.

a Regression coefficients (β) and 95% CI were estimated from linear regression models. Shape parameters were scaled with Z-standardization (($X\;–\;\bar{X}$)/standard deviation). Volume was natural log-transformed and elongation was transformed with a Box-Cox transformation.

b Interpretation: aneurysm volume was 0.33 standard deviations smaller in women compared to men.

c Values were multiplied by 100 to improve readability.

d Odds ratios and 95% CI were estimated from logistic regression models. Morphological parameters were dichotomized based on the median and entered as dependent variable, with above-median values coded as 1.

e Interpretation: the odds of an above-median value for shape index in women was 2.38 times higher compared to men.

**Fig. 3:**
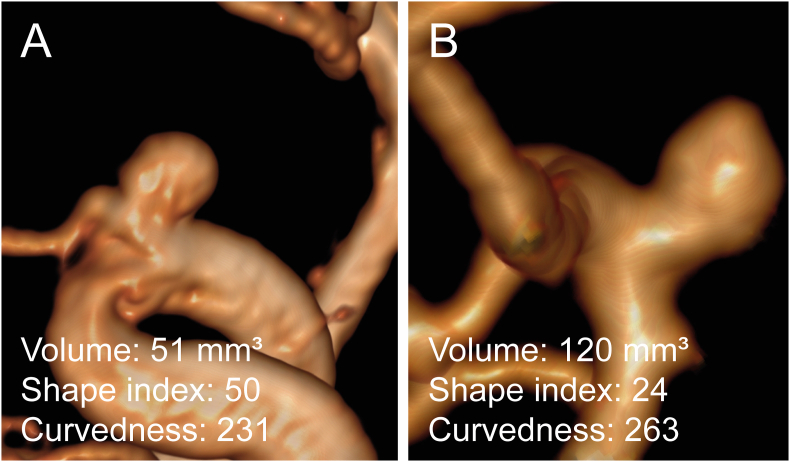
Example unruptured intracranial aneurysms in a woman and a man. Multiplanar reconstructions of unruptured intracranial aneurysms. **a** 34-year old woman with 4-mm aneurysm of the right internal carotid artery on MR angiography. **b** 52-year old man with 7-mm aneurysm of the left internal carotid artery on CT angiography. Aneurysms in women generally had lower volumes, higher shape index, and lower curvedness values compared to men. Sphericity, shape index, and curvedness were multiplied by 100.

The first sensitivity analysis in which smoking was removed from the multivariable analysis yielded similar results (Supplemental Table 1). The second sensitivity analysis in which only the subset of patients with complete data for smoking status was used (319 patients, 233 women [73%]) also yielded similar results (Supplemental Table 2). Example aneurysms in a woman and a man are displayed in Fig. 3.

## 4 Discussion

By using 3D assessment of intracranial aneurysm morphology, we found sex differences in aneurysm volume and shape. Aneurysms in women generally had lower volumes, higher shape index, and lower curvedness values compared to men—characteristics associated with a lower risk of rupture.

Most previous studies on sex differences in aneurysm size and shape did not find statistically significant differences (Krzyżewski et al., 2018; Juchler et al., 2020; Wälchli et al., 2024). However, the trends observed in these studies are in line with our findings. One study that assessed sex differences in aneurysm characteristics in 1484 ruptured and unruptured aneurysms found that aneurysms in women were slightly smaller (5.6 vs 5.9 mm) and, in a subset of 48 aneurysms, less often irregular (59% vs 64%) (Wälchli et al., 2024). Similarly, a study evaluating sex differences in 526 ruptured and unruptured aneurysms found that aneurysms in women had a smaller size (6.0 vs 6.7 mm) and volume (345 vs 437 mm^3^) than aneurysms in men (Krzyżewski et al., 2018). Another study assessed factors associated with 3D quantified morphological parameters in 134 ruptured and unruptured aneurysms and found a trend toward a smaller non-sphericity index in women (Juchler et al., 2020), consistent with our univariable analysis for sphericity, showing a larger sphericity in women. Last, a study of 474 aneurysms found a statistically significant sex difference in aneurysm size: women had significantly smaller aneurysm sizes compared to men (2.1 vs 2.9 mm). However, this study included only ruptured anterior communicating artery aneurysms (Lin et al., 2016).

Our study adds to previous research by utilizing 3D quantified morphological parameters instead of relying on visual assessments, allowing for a more precise evaluation of sex differences in aneurysm morphology. This may explain why we found statistically significant sex differences in contrast to previous studies. Another important addition to the existing literature is that we included only unruptured aneurysms, whereas most prior studies evaluated both ruptured and unruptured aneurysms together. Since rupture itself may alter aneurysm morphology (Kamphuis et al., 2024), this could have homogenized the differences between women and men in aneurysm morphology in previous studies.

In the main analysis, aneurysm shape index was higher in women compared to men. In the sensitivity analyses, aneurysm shape index remained higher in women than in men. This reinforces the conclusion that the observed sex differences in aneurysm morphology are unlikely to be substantially confounded by smoking status.

Exploratory analyses in which shape index and curvedness were analyzed as continuous variables largely supported the findings of the primary categorical analyses. For curvedness, the direction and significance of the association were consistent across both approaches, with women showing lower curvedness than men after multivariable adjustment. For shape index, the association observed in the categorical analysis was attenuated in the continuous multivariable model. This attenuation may reflect the limited variability of this parameter, which can reduce the sensitivity of linear models to detect small between-sex differences.

An important implication of our results is that future cohort studies investigating the morphological predictors of aneurysm instability should stratify or adjust for sex to improve the accuracy of aneurysm risk prediction. The relationship between morphology characteristics and the risk of aneurysm instability could be different for women and men (effect modification), or it could be confounded by sex.

Since morphology of aneurysms in women point to a lower risk of rupture compared to those in men, other factors than aneurysm morphology should explain the higher aneurysmal rupture risk in women. One possibility is that this increased risk is related to sex hormones, as studies suggest that women are more prone to SAH after menopause (Kongable et al., 1996; Etminan et al., 2019; Lai et al., 2022). Another explanation is that women may experience higher hemodynamic forces on the vessel wall. Research has shown that the diameter of most vessels in the circle of Willis is smaller in women than in men (Lindekleiv et al., 2010; Mujagić et al., 2013; Shatri et al., 2017; Groenheide et al., 2025), potentially leading to higher blood flow velocity and greater hemodynamic stress (Lindekleiv et al., 2010). A third explanation is that women and men may respond differently to environmental risk factors. For instance, it has been suggested that smoking increases the risk of aneurysmal SAH more in women than in men (Lindekleiv et al., 2011). Last, the difference in rupture risk could be influenced by sex-specific genetic risk factors, including genetic risk factors of the X-chromosome (Zuurbier et al., 2022). This needs to be studied in future research.

A strength of our study is the use of 3D quantified morphological parameters to assess aneurysm volume and shape, enabling a more precise evaluation of morphological differences compared to visual assessment. Another strength is that we included only UIAs, which is more relevant for predicting rupture risk, as rupture itself could lead to morphological changes (Kamphuis et al., 2024).

We also need to address two limitations. One limitation of our study is the potential for selection bias. Women may be more likely than men to undergo screening for UIAs if they have a positive family history of SAH (Bor et al., 2014). If not detected through screening, UIAs are typically detected during the diagnostic workup for cardiovascular disease. As a result, comorbidities are relatively more prevalent in men than in women with UIAs. Consistent with this, hypertension was more common among men in our sample. Since hypertension is associated with a rupture-prone aneurysm morphology (Liu et al., 2019), this could have led to an overestimation of rupture-prone aneurysm morphology in men. We addressed this by adjusting for hypertension in the multivariable analysis. Another limitation is that our study had a cross-sectional design, which did not allow us to examine the association between sex-specific aneurysm morphology and long-term aneurysm instability.

## 5 Conclusion

By using 3D assessment of intracranial aneurysm morphology, we identified sex differences in aneurysm volume and shape. This emphasizes the need to stratify or adjust for sex in future research on morphological predictors of aneurysm instability. Aneurysm morphology characteristics in women are associated with a lower risk of rupture, yet women have a higher risk of aneurysm rupture. This suggests that other factors than aneurysm morphology must explain the increased risk in women. Future cohort studies should investigate potential explanatory factors, such as smoking, sex hormones, hemodynamics, or genetic predispositions.

Data and code availability statement.

Anonymized data and R scripts not published within this article will be made available by request from any qualified investigator.

## CRediT authorship contribution statement

**Maarten J. Kamphuis:** Writing – original draft, Visualization, Methodology, Investigation, Formal analysis, Data curation, Conceptualization. **Phebe J. Groenheide:** Writing – review & editing, Visualization. **Laura T. van der Kamp:** Writing – review & editing, Data curation. **Margot van Genderen:** Data curation. **Ruben P.A. van Eijk:** Methodology. **Jeroen Hendrikse:** Writing – review & editing. **Gabriel J.E. Rinkel:** Writing – review & editing, Data curation. **Mervyn D.I. Vergouwen:** Writing – review & editing, Data curation. **Ynte M. Ruigrok:** Writing – review & editing, Supervision, Conceptualization. **Irene C. van der Schaaf:** Writing – review & editing, Supervision, Conceptualization.

## Funding

Prof. Dr. Irene van der Schaaf has received funding from TKI-LSH Health Holland. Prof. Dr. Ynte M. Ruigrok has received funding from the European Research Council (ERC) under the European Union's Horizon 2020 research and innovation program (grant agreement No. 852173) and a Clinical Established Investigator Grant by the Dutch Heart Foundation (Dekker grant 03–001–2022–0157). Dr. Mervyn D.I. Vergouwen is supported by a Clinical Established Investigator Grant by the Dutch Heart Foundation (Dekker grant 2018T076).

## Declaration of competing interest

Declarations of competing interest: none.

## Data Availability

Data will be made available on request.
