# Supplementary material for: Sex differences in the three-dimensional morphology of unruptured intracranial aneurysms
Source: Neuroimage Rep. 2026 Feb 16;6(1):100328. doi: 10.1016/j.ynirp.2026.100328 (PMC12925532; doi:10.1016/j.ynirp.2026.100328)
Supplement: Multimedia component 2 [file mmc2.pdf]

SUPPLEMENTAL MATERIALS

Sex differences in the three-dimensional morphology of unruptured intracranial aneurysms

Supplemental Figure 1. Distribution of 3D quantified morphological parameters.

Supplemental Figure 2. Q-Q plots of 3D quantified morphological parameters.

Supplemental Figure 3. Q-Q plots of residuals from multivariable linear regression models.

Supplemental Figure 4. Three-dimensional quantified morphological parameters stratified by sex.

Supplemental Table 1. Multivariable women-to-men differences in 3D quantified morphological parameters, without smoking as a covariable.

Supplemental Table 2. Women-to-men differences in 3D quantified morphological parameters for a subset of patients with complete data for smoking.

Supplemental Figure 1. Distribution of 3D quantified morphological parameters.

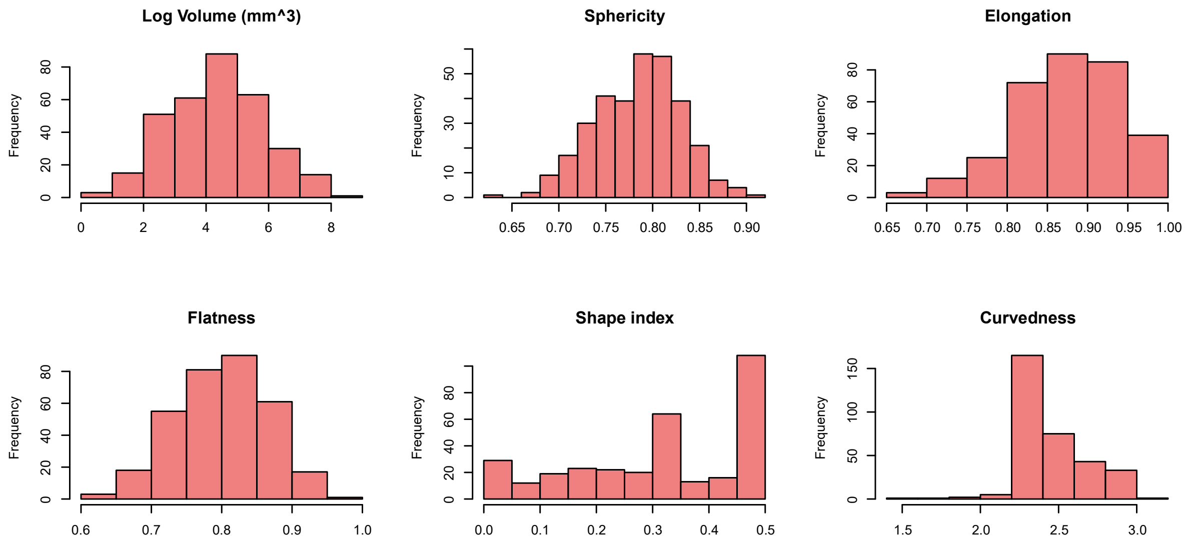

Performed on the single merged imputed dataset.

Shape parameters were scaled with Z-standardization ((X – X̄)/SD). Volume was natural log-transformed and elongation was transformed with a Box-Cox transformation.

Supplemental Figure 2. Q-Q plots of 3D quantified morphological parameters.

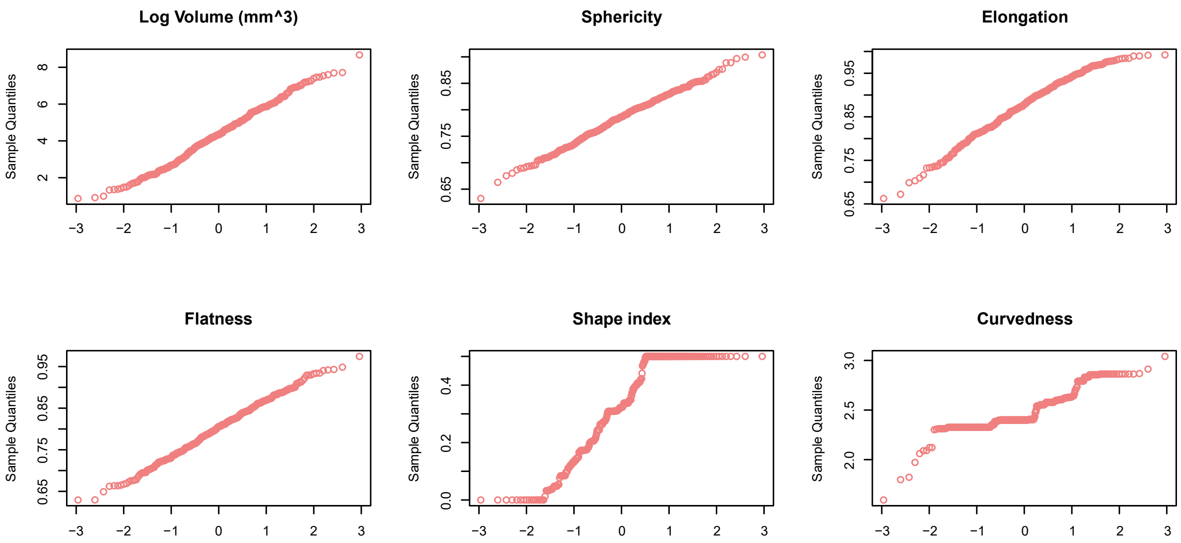

Performed on the single merged imputed dataset.

Shape parameters were scaled with Z-standardization ((X – X̄)/SD). Volume was natural log-transformed and elongation was transformed with a Box-Cox transformation.

Supplemental Figure 3. Q-Q plots of residuals from multivariable linear regression models.

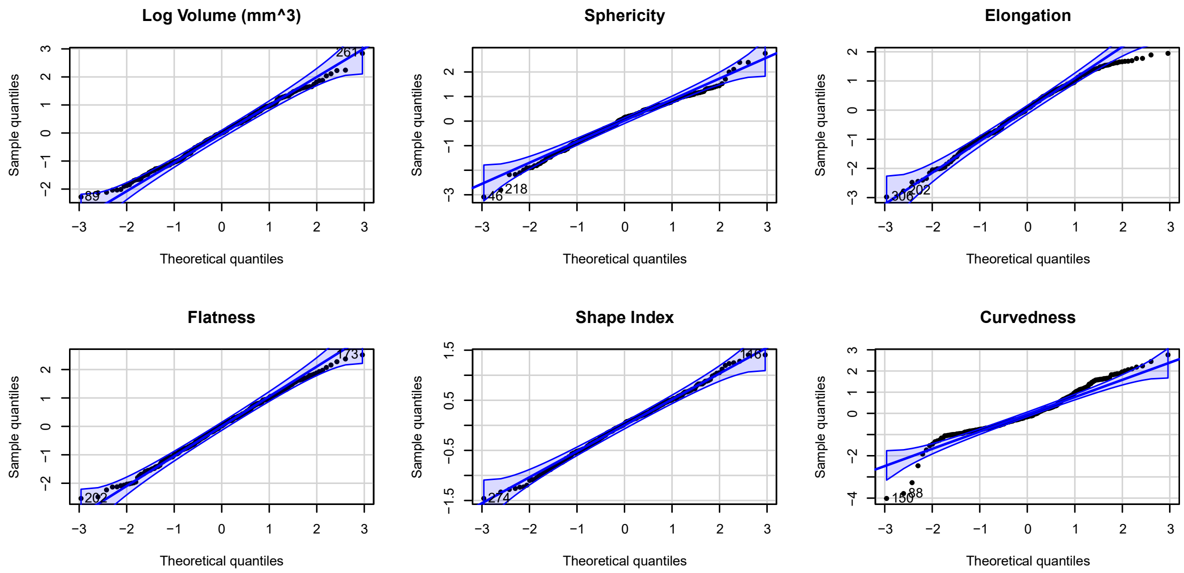

Performed on the single merged imputed dataset.

Morphological parameters were used as dependent variables; sex and confounders (age, hypertension, smoking status, aneurysm size, aneurysm location, and imaging modality) as independent variables. Volume was not adjusted for aneurysm size. Men were used as reference.

Shape parameters were scaled with Z-standardization ($(X - \bar{X})/SD$). Volume was natural log-transformed and elongation was transformed with a Box-Cox transformation.

Supplemental Figure 4. Three-dimensional quantified morphological parameters stratified by sex.

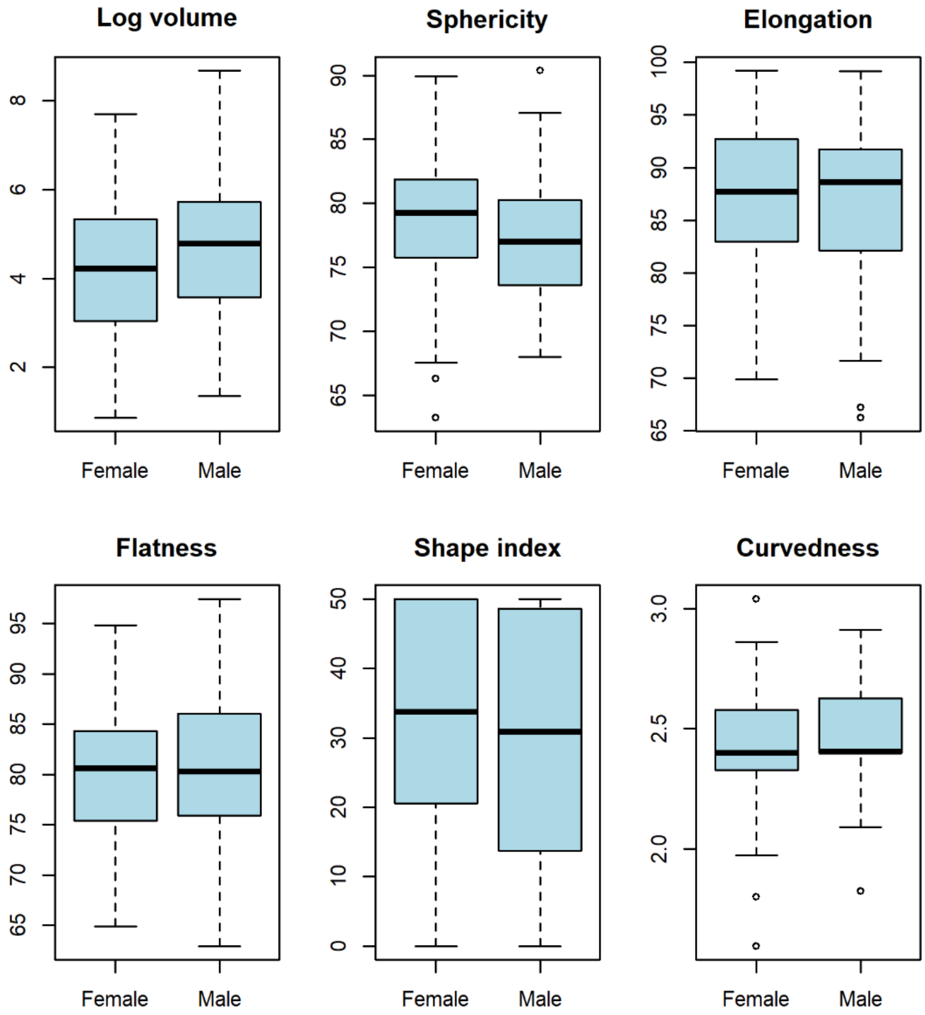

Sphericity, elongation, flatness, and shape index were multiplied by 100. Volume was natural log-transformed.

Supplemental Table 1. Multivariable women-to-men differences in 3D quantified morphological parameters, without smoking as a covariable.

**Global shape parameters**	Multivariable<br>Women-to-men difference<br>(β, 95% CI)[a]	
Volume (mm^3)	**-0.31 (-0.55 to -0.06)**	
Sphericity[b]	0.20 (-0.02 to 0.43)	
Elongation[b]	0.04 (-0.22 to 0.30)	
Flatness[b]	-0.03 (-0.28 to 0.22)	
**Local shape parameters**	Multivariable<br>Women-to-men difference<br>(β, 95% CI)[a]	Multivariable<br>Women-to-men difference<br>(odds ratio, 95% CI)[c]
Shape index[c]	0.07 (-0.07 to 0.20)	**2.38 (1.03 to 5.49)**
Curvedness[c]	**-0.27 (-0.50 to -0.03)**	**0.50 (0.29 to 0.88)**

Morphological parameters were used as dependent variables; sex and confounders (age, hypertension, aneurysm size, aneurysm location, and imaging modality) as independent variables. Men were used as reference. Volume was not adjusted for aneurysm size. Statistically significant associations indicated in bold.

[a] Regression coefficients (β) and 95% CI were estimated from linear regression models. Shape parameters were scaled with Z-standardization ($(X - \bar{X})$/standard deviation). Volume was natural log-transformed and elongation was transformed with a Box-Cox transformation.

[b] Values were multiplied by 100 to improve readability.

[c] Odds ratios and 95% CI were estimated from logistic regression models. Morphological parameters were dichotomized based on the median and entered as dependent variable, with above-median values coded as 1.

Supplemental Table 2. Women-to-men differences in 3D quantified morphological parameters for a subset of patients with complete data for smoking.

Global shape parameters

	Median (interquartile range)			Women-to-men difference (β, 95% CI)[a]	
	Total (n=319)	Women (n=233, 73%)	Men (n=86, 27%)	Univariable	Multivariable
Volume (mm^3)	77 (27–252)	69 (21–216)	120 (39–308)	**-0.33 (-0.58 to -0.09)[b]**	**-0.30 (-0.54 to -0.06)**
Sphericity[c]	79 (76–81)	79 (76–82)	77 (74–80)	**0.38 (0.14 to 0.62)**	0.20 (-0.02 to 0.43)
Elongation[c]	88 (83–93)	88 (83–93)	88 (82–92)	0.05 (-0.19 to 0.30)	0.04 (-0.22 to 0.30)
Flatness[c]	81 (76–85)	81 (75–84)	81 (76–86)	-0.08 (-0.32 to 0.17)	-0.03 (-0.29 to 0.23)

Local shape parameters

	Median (interquartile range)			Women-to-men difference (β, 95% CI)[a]		Women-to-men difference (odds ratio, 95% CI)[d]	
	Total (n=319)	Women (n=233)	Men (n=86)	Univariable	Multivariable	Univariable	Multivariable
Shape index[c]	32 (19–50)	34 (21–50)	31 (13–46)	**0.36 (0.12 to 0.61)**	0.06 (-0.07 to 0.20)	**2.38 (1.44 to 4.01)[e]**	**2.38 (1.05 to 5.61)**
Curvedness[c]	240 (235–260)	240 (233–258)	240 (240–262)	-0.20 (-0.44 to 0.05)	**-0.27 (-0.51 to -0.03)**	0.67 (0.41 to 1.09)	**0.56 (0.32 to 0.95)**

Morphological parameters were used as dependent variables; sex and confounders (age, hypertension, smoking status, aneurysm size, aneurysm location, and imaging modality) as independent variables. Volume was not adjusted for aneurysm size. Men were used as reference. Statistically significant associations indicated in bold.

[a] Regression coefficients (β) and 95% CI were estimated from linear regression models. Shape parameters were scaled with Z-standardization ($(X - \bar{X})/SD$). Volume was natural log-transformed and elongation was transformed with a Box-Cox transformation.

[b] Interpretation: aneurysm volume was 0.33 standard deviations smaller in women compared to men.

[c] Values were multiplied by 100 to improve readability.

[d] Odds ratios and 95% CI were estimated from logistic regression models. Morphological parameters were split by the median and entered as dependent variable, with above-median values coded as 1.

[e] Interpretation: the odds of an above-median value for shape index in women was 2.38 times higher compared to men.
